# *PODXL2* Expression Is Associated with Inferior Survival, Adverse-Risk Features, and Distinct Transcriptional Programs in Acute Myeloid Leukemia

**DOI:** 10.3390/cancers18193219

**Published:** 2026-10-06

**Authors:** Hyun Min Ko, Katherine Scarton, Samantha C. Covey, Lucio Miele, Sun Young Kim

**Affiliations:** 1 School of Medicine, LSU Health Science Center, New Orleans, LA 70112, USA; hko@lsuhsc.edu (H.M.K.); kscart@lsuhsc.edu (K.S.);; 2 Department of Microbiology, Genetics and Immunology, LSU LCMC Health Cancer Center, New Orleans, LA 70112, USA; 3 Department of Interdisciplinary Oncology, LSU LCMC Health Cancer Center, New Orleans, LA 70112, USA

**Keywords:** AML, E2F, mTORC1, MYC, *PODXL2*, *TP53*

## Abstract

Background: Acute myeloid leukemia (AML) is characterized by substantial biological heterogeneity that is incompletely captured by current risk-classification frameworks. Podocalyxin-like 2 (*PODXL2*), a CD34-family transmembrane sialomucin implicated in tumor progression in solid tumors, remains poorly characterized in AML. Methods: We conducted integrated clinical, genomic, transcriptomic, regulatory network, and *ex vivo* drug-response analyses using two independent AML cohorts, TCGA-LAML and BeatAML. Results: Elevated *PODXL2* expression was consistently associated with inferior overall survival across both cohorts, including after adjustment for established clinical risk factors. *PODXL2*-high AML was enriched for adverse-risk features, including ELN 2022 adverse-risk classification, poor-risk cytogenetics, and *TP53* mutations, whereas *IDH2* mutations were more frequent in *PODXL2*-low AML. Transcriptomic analyses identified a distinct *PODXL2*-high state characterized by enrichment of MYC and E2F target programs, cell-cycle progression, DNA repair, oxidative phosphorylation, mTORC1 signaling, and metabolic pathways. Regulatory network analyses demonstrated enhanced MYC/E2F-associated regulatory activity. *PODXL2*-high AML also exhibited altered differentiation-state features, characterized by reduced representation of monocyte-like and conventional dendritic cell-like malignant cell states. *Ex vivo* drug-response profiling identified distinct *PODXL2*-associated response patterns, including reduced sensitivity to OTX-015 and SRC-family kinase inhibitors. Conclusions: *PODXL2* expression is associated with adverse clinical and molecular features, distinct transcriptional programs, altered differentiation states, and differential *ex vivo* drug-response patterns in AML. These findings identify *PODXL2* expression as a marker associated with adverse clinical and molecular features and inferior survival in AML, although its prognostic discrimination as a standalone marker is modest. Further functional and prospective studies are warranted to establish its biological and clinical significance.

## Simple Summary

Acute myeloid leukemia (AML) is a biologically diverse blood cancer with markedly different outcomes among patients. Current risk-classification systems do not fully capture this variability, highlighting the need for additional biomarkers that may refine risk stratification and prognostic assessment. In this study, we investigated the expression of podocalyxin-like 2 (*PODXL2*), which has been linked to cancer progression in several solid tumors but has not been well studied in AML. By analyzing two large patient datasets, we found that higher *PODXL2* expression was associated with poorer survival, adverse genetic features, distinct biological characteristics, and differences in responses to certain anticancer drugs. These findings suggest that elevated *PODXL2* expression is associated with adverse disease characteristics in AML, although its ability to improve prognostic prediction beyond established clinical risk factors appears limited. Further laboratory and clinical studies are needed to determine the biological and clinical significance of *PODXL2* in AML.

## 1. Introduction

Acute myeloid leukemia (AML) is a biologically heterogeneous hematologic malignancy characterized by diverse genetic alterations, variable clinical behavior, and substantial differences in treatment response and survival outcomes [1,2,3]. Although current classification and risk-stratification systems incorporate recurrent cytogenetic and molecular abnormalities, considerable heterogeneity remains among patients with similar genomic profiles [4,5,6]. These observations suggest that genomic alterations alone do not fully capture the biological programs and cellular states that contribute to disease aggressiveness and clinical outcome [4,7,8].

Transcriptomic profiling provides a complementary framework for characterizing AML heterogeneity because gene-expression patterns reflect the integrated consequences of genetic alterations, epigenetic regulation, signaling activity, and cellular differentiation state [9,10,11]. Previous studies have demonstrated that transcriptional programs associated with stemness, proliferation, metabolic adaptation, and therapeutic resistance are linked to AML biology and clinical outcomes, indicating that gene-expression profiles may provide clinically relevant information beyond individual genomic alterations [7,9,11,12,13].

Podocalyxin-like 2 (PODXL2), also known as endoglycan, is a member of the CD34 family of transmembrane sialomucins, which includes CD34 and podocalyxin (PODXL) [14,15]. Members of this family play important roles in hematopoietic development, vascular biology, cell adhesion, migration, and signal transduction [14,15,16,17]. While PODXL has been extensively studied in cancer and implicated in tumor progression, therapeutic resistance, and adverse clinical outcomes across multiple malignancies, the biological and clinical significance of PODXL2 remains less well understood [18,19].

Emerging evidence suggests that PODXL2 may function as more than a passive cell-surface marker. Increased *PODXL2* expression has been associated with aggressive disease phenotypes across several solid tumors, including prostate cancer, breast cancer, and cholangiocarcinoma [20,21,22,23]. Functional studies have implicated PODXL2 in the regulation of cellular survival, proliferation, stemness, and tumor progression, through the PI3K/AKT, Rac1/AKT, and MAPK/ERK signaling pathways [20,21,22]. Given the established roles of these pathways in AML pathogenesis, *PODXL2* expression may identify a biologically distinct subset of AML characterized by enhanced proliferative and metabolic activity [24,25,26]. Furthermore, because these signaling pathways converge on transcriptional regulators such as MYC and E2F, *PODXL2* expression may be associated with distinct characteristic transcriptional programs relevant to AML progression [24,27,28,29].

Despite these observations, the clinical and biological significance of *PODXL2* expression in AML remains poorly understood. Although previous integrative genomic studies have implicated *PODXL2* in the molecular landscape of AML, these investigations were largely exploratory and did not determine whether *PODXL2* provides prognostic information independent of established cytogenetic and molecular risk factors [30]. In particular, it remains unclear whether *PODXL2* expression identifies a distinct AML state associated with adverse outcomes, characteristic transcriptional programs, and specific regulatory network activity.

We therefore hypothesized that elevated *PODXL2* expression is associated with a biologically distinct AML phenotype characterized by adverse clinical outcomes and unique transcriptional and regulatory features. To test this hypothesis, we performed integrated analyses of clinical outcomes, recurrent genomic alterations, transcriptomic profiles, pathway activity, transcription-factor regulatory networks, and *ex vivo* drug-response data across two independent AML cohorts, the Cancer Genome Atlas AML (TCGA-LAML) and BeatAML. Our objective was to determine the prognostic significance of *PODXL2* expression and characterize the genomic, molecular, transcriptional, and regulatory features associated with elevated *PODXL2* expression in AML.

## 2. Methods

### 2.1. Study Cohorts and Data Acquisition

Transcriptomic, genomic, and clinical data were analyzed from two independent AML cohorts, TCGA-LAML and BeatAML. TCGA-LAML data were obtained from publicly available TCGA resources and the original TCGA AML study [31]. BeatAML transcriptomic, genomic, clinical, and *ex vivo* drug-response data were obtained from the publicly available BeatAML dataset [32]. The two cohorts were analyzed independently throughout the study and were not pooled for statistical analyses.

For TCGA-LAML, RNA-sequencing data were matched with available clinical, cytogenetic, and molecular annotations, yielding a final transcriptomic cohort of 151 patients. For BeatAML, analyses were restricted to specimens collected at initial diagnosis. Clinical records were matched to RNA-sequencing samples using unique sample identifiers, and specimens obtained outside the initial diagnostic setting were excluded. When multiple eligible specimens were available for a patient, a single specimen was selected according to a predefined hierarchy prioritizing bone marrow aspirate, followed by peripheral blood and leukapheresis specimens. If multiple specimens shared the same priority level, the sample collected closest to study enrollment was selected. This process generated a non-redundant BeatAML transcriptomic cohort of 448 patients, of whom 444 had evaluable overall survival (OS) data after exclusion of cases with missing survival information.

Within each cohort, patients were stratified into *PODXL2*-low and *PODXL2*-high groups based on the cohort-specific median *PODXL2* expression level. In BeatAML, the median was calculated after restricting the dataset to initial-diagnosis samples and selecting one specimen per patient. This cohort-specific median dichotomization was used for exploratory within-cohort group comparisons and was not intended to define a clinically transferable *PODXL2* expression threshold. Because data availability differed across clinical, genomic, survival, transcriptomic, and *ex vivo* drug-response datasets, individual analyses were restricted to samples with the data required for the corresponding analysis after dataset-specific matching and filtering. Accordingly, evaluable sample sizes varied across analyses, and exact sample sizes are reported in the corresponding figure legends.

### 2.2. Clinical and Molecular Characteristics

Clinical and molecular annotations were curated within each cohort prior to analysis. Baseline characteristics included age, sex, race, white blood cell (WBC) count, bone marrow blast percentage, peripheral blood blast percentage, cytogenetic features, French-American-British (FAB) classification, and recurrent AML-associated molecular alterations, when available.

FAB classifications were standardized according to the M0-M7 categories. Analyses involving FAB classification were restricted to patients with available FAB data. No FAB categories were excluded based on sample size. Category-specific sample sizes, including those of rare FAB subtypes, are reported in the corresponding figures.

### 2.3. European LeukemiaNet (ELN) 2022 Risk Classification

ELN 2022 risk categories were reconstructed independently for the TCGA-LAML and BeatAML cohorts using available cytogenetic, fusion, and molecular data. For TCGA-LAML, cytogenetic and fusion annotations were integrated with somatic mutation data. Gene-level Allele-Specific Copy-Number Analysis of Tumors (ASCAT) copy-number data were used for quality-control purposes and were not considered a substitute for conventional karyotype information. For BeatAML, curated clinical and molecular annotations were integrated with available baseline whole-exome sequencing (WES) and targeted sequencing data. Sequencing-dependent alterations were evaluated only when corresponding mutation-call data were available.

Risk assignment was based on the ELN 2022 classification framework and incorporated core-binding factor abnormalities (*RUNX1::RUNX1T1* and *CBFB::MYH11*), *CEBPA* bZIP in-frame mutations, *NPM1* and *FLT3-ITD* status, *TP53* mutations, myelodysplasia-related gene mutations (*ASXL1*, *BCOR*, *EZH2*, *RUNX1*, *SF3B1*, *SRSF2*, *STAG2*, *U2AF1*, and *ZRSR2*), and ELN-defined cytogenetic abnormalities. *TP53* mutations with a variant allele frequency (VAF) ≥10% were classified as adverse-risk alterations consistent with ELN 2022 recommendations [5]. Acute promyelocytic leukemia (APL) was identified separately based on available evidence of *PML::RARA* or t(15;17).

Risk categories were assigned according to the hierarchical framework outlined in the ELN 2022 recommendations [5]. Cases harboring favorable-defining core-binding factor abnormalities or *CEBPA* bZIP in-frame mutations were classified as favorable risk. *NPM1*-mutated/*FLT3*-ITD-negative AML was classified as favorable risk in the absence of adverse-risk cytogenetic abnormalities. *MLLT3::KMT2A*/t(9;11) AML was classified as intermediate risk, irrespective of concurrent myelodysplasia-related gene mutations. Cases harboring ELN-defined adverse-risk cytogenetic or molecular abnormalities were classified as adverse risk. Remaining *FLT3*-ITD-positive cases without higher-priority defining abnormalities were classified as intermediate risk. Cases lacking sufficient information for definitive ELN assignment were classified as unresolved and excluded from analyses requiring ELN risk categorization.

### 2.4. Survival Analysis

OS was analyzed independently in the TCGA-LAML and BeatAML cohorts. Kaplan-Meier survival curves were generated for *PODXL2*-low and *PODXL2*-high groups and compared using the log-rank test. Cox proportional-hazards regression was used to estimate hazard ratios (HRs) and 95% confidence intervals (CIs).

Univariable Cox regression models evaluated *PODXL2* expression as both a categorical variable (*PODXL2*-high versus *PODXL2*-low) and a continuous variable. Continuous *PODXL2* expression was standardized within each cohort and modeled per 1-standard deviation (SD) increase. Age was modeled per 10-year increase. Multivariable Cox regression models included *PODXL2* expression group, age, sex, and ELN 2022 adverse-risk status (adverse versus non-adverse). The proportional-hazards assumption was formally evaluated using tests based on scaled Schoenfeld residuals. Because evidence of non-proportional hazards was observed in the BeatAML model, a sensitivity Cox model incorporating interactions with log-transformed follow-up time was additionally fitted for covariates showing evidence of non-proportionality.

Time-dependent receiver operating characteristic (ROC) analyses were performed to evaluate the prognostic performance of *PODXL2* expression at 12, 24, and 36 months.

### 2.5. Genomic Alteration Analysis

Associations between *PODXL2* expression and recurrent AML-associated genomic alterations were evaluated independently in the TCGA-LAML and BeatAML cohorts. Analyses included *TP53*, *FLT3*-ITD, *NPM1*, *RUNX1*, *ASXL1*, *CEBPA* bZIP in-frame mutations, myelodysplasia-related gene mutations, and complex karyotypes, when evaluable.

Alteration frequencies were compared between *PODXL2*-low and *PODXL2*-high groups using two-sided Fisher’s exact tests. Odds ratios (ORs) and 95% CIs were calculated to estimate the strength and direction of associations. Multiple-testing correction was performed using the Benjamini-Hochberg (BH) procedure.

### 2.6. Association with Clinical, Cytogenetic and Morphologic Characteristics

Associations between *PODXL2* expression and clinical, cytogenetic, and morphologic characteristics were evaluated independently in the TCGA-LAML and BeatAML cohorts. ELN 2022 risk, cytogenetic risk, and FAB classification were compared between *PODXL2*-low and *PODXL2*-high groups when corresponding annotations were available.

Differences in categorical characteristics between *PODXL2*-low and *PODXL2*-high groups were assessed using two-sided Fisher’s exact tests. Continuous clinical variables were summarized using medians and compared using two-sided Wilcoxon rank-sum tests.

Associations between continuous *PODXL2* expression and ELN 2022 risk category, cytogenetic risk group, and FAB classification were evaluated using Kruskal–Wallis tests. When appropriate, pairwise comparisons were subsequently performed using Dunn’s multiple-comparison tests with BH correction. Only patients with evaluable annotations for the characteristic under investigation were included in the corresponding analyses.

### 2.7. Malignant Cell-Type Signature Analysis

Malignant cell-type signature scores for BeatAML samples were obtained from the BeatAML 2.0 resource [32]. The analyzed signatures included hematopoietic stem cell (HSC)-like, progenitor-like, granulocyte-monocyte progenitor (GMP)-like, promonocyte-like, monocyte-like, and conventional dendritic cell (cDC)-like programs derived from previously reported single-cell RNA-sequencing–based AML cell-state signatures [33]. Signature scores were matched to the selected initial-diagnosis BeatAML RNA-sequencing samples using sample identifiers and transformed to *z*-scores. Standardization was performed separately for each cell-state signature.

Differences in signature scores between *PODXL2*-low and *PODXL2*-high groups were assessed using two-sided Wilcoxon rank-sum tests, with BH correction applied across the six signatures. Associations between FAB categories (M0–M7) and malignant cell-state signature scores were evaluated using point-biserial correlation analysis, with each FAB category encoded as a binary variable. Multiple testing across FAB-signature associations was controlled using the BH procedure.

### 2.8. Differential Gene-Expression Analysis

Transcriptomic analyses were performed independently in the TCGA-LAML and BeatAML cohorts. Differential gene expression between *PODXL2*-low and *PODXL2*-high AML samples was assessed using the limma framework. Differentially expressed genes (DEGs) were identified using empirical Bayes moderation and defined as genes with a false discovery rate (FDR) < 0.05 and an absolute log_2_ fold change ≥ 0.5.

### 2.9. Pathway-Enrichment Analysis

Gene-set enrichment analysis (GSEA) was performed using Hallmark gene sets from the Molecular Signatures Database (MSigDB) to identify biological pathways associated with differential gene expression between *PODXL2*-low and *PODXL2*-high AML samples. GSEA was implemented using the fgseaMultilevel algorithm, with gene-set sizes restricted to 10–500 genes and eps = 0. Because the multilevel algorithm was used, a fixed number of permutations was not specified. Enrichment was quantified using normalized enrichment scores (NES), and multiple-testing correction was performed using the BH procedure.

Sample-level Hallmark pathway activity was additionally estimated using gene-set variation analysis (GSVA) with a Gaussian kernel and gene-set sizes restricted to 10–500 genes. Associations between continuous *PODXL2* expression and GSVA-derived pathway-activity scores were evaluated using Spearman rank correlation, with BH correction applied for multiple comparisons.

### 2.10. Transcription-Factor Activity Analysis

Transcription-factor activity was inferred from transcriptomic profiles using Discriminant Regulon Expression Analysis (DoRothEA) regulons and the Virtual Inference of Protein-activity by Enriched Regulon analysis (VIPER) algorithm implemented through decoupleR. Human DoRothEA regulons were used, retaining interactions with CIs A, B, or C, and only regulons containing at least five measured target genes were included. Differential transcription-factor activity between *PODXL2*-low and *PODXL2*-high AML samples was evaluated independently in the TCGA-LAML and BeatAML cohorts. Multiple-testing correction was performed using the BH procedure.

### 2.11. Ex Vivo Drug-Response Analysis

*Ex vivo* drug-response analyses were performed using BeatAML data. Drug-response measurements were matched to the corresponding selected initial-diagnosis transcriptomic samples using subject and RNA-sequencing sample identifiers. Analyses were restricted to compounds with evaluable responses in at least 180 specimens to exclude sparsely measured compounds and maintain adequate sample coverage across the drug-response analyses. This threshold was used as a data-coverage criterion rather than being derived from a formal statistical power calculation. Following application of this criterion, 113 compounds were included in the drug-response analysis. Drug response was quantified using the area under the dose-response curve (AUC), with lower AUC values indicating greater drug sensitivity. For cross-drug visualization, AUC values were standardized to z-scores separately for each drug, with lower z-scores indicating greater relative *ex vivo* sensitivity. Differences in drug response between *PODXL2*-low and *PODXL2*-high AML samples were assessed using two-sided Wilcoxon rank-sum tests. Associations between continuous *PODXL2* expression and drug response were evaluated using Spearman rank correlation. Multiple-testing correction was performed using the BH procedure.

### 2.12. Statistical Analysis and Use of Artificial Intelligence (AI)

Unless otherwise specified, all analyses were performed in R version 4.0.3 (R Foundation for Statistical Computing, Vienna, Austria). All statistical tests were two-sided. Continuous variables were analyzed using nonparametric tests and categorical variables using Fisher’s exact tests, as appropriate. Comparisons involving more than two groups were evaluated using Kruskal–Wallis tests followed, when appropriate, by Dunn’s multiple-comparison tests.

Correlation methods were selected according to the analysis. Spearman rank correlation was used to evaluate associations among continuous transcriptomic, pathway-activity, and drug-response measures, whereas point-biserial correlation was used to assess associations between binary FAB subtype membership and continuous malignant cell-state signature scores.

Multiple-testing correction was performed using the BH procedure. Unless otherwise specified, a BH-adjusted *q*-value < 0.05 was considered statistically significant. For *ex vivo* drug-response analyses, a BH-adjusted *q*-value < 0.10 was prespecified as an exploratory threshold given the hypothesis-generating nature of this analysis. This threshold was used to identify candidate drug-response associations for further investigation rather than to establish definitive or independently validated drug-response biomarkers.

Microsoft Copilot (free/basic version, GPT-based conversational AI assistant) and ChatGPT (free/basic version, OpenAI conversational AI assistant) were used to improve English fluency, grammar, sentence structure, clarity, and readability of the text. The AI tools were not used for data analysis, interpretation of results, generation of research findings, or scientific decision-making. All content was reviewed and verified by the author.

## 3. Results

### 3.1. PODXL2 Expression Is Associated with Inferior Survival in AML

To evaluate the prognostic significance of *PODXL2* expression, OS was analyzed independently in the TCGA-LAML and BeatAML cohorts. *PODXL2*-high AML was associated with significantly inferior OS in both TCGA-LAML and BeatAML (log-rank *p* = 0.0035 and 0.0114, respectively; Figure 1a,b).

Consistent with the Kaplan-Meier analyses, univariable Cox regression models demonstrated that higher *PODXL2* expression was associated with inferior OS when evaluated as a continuous variable in both TCGA-LAML (HR per 1-SD increase, 1.25; 95% CI, 1.05–1.48; *p* = 0.011) and BeatAML (HR per 1-SD increase, 1.21; 95% CI, 1.07–1.37; *p* = 0.003) (Figure 1c,d; Table 1). Consistent associations were observed using the cohort-specific median-based classification, with *PODXL2*-high expression associated with an increased risk of death in TCGA-LAML (HR, 1.76; 95% CI, 1.20–2.58; *p* = 0.004) and BeatAML (HR, 1.38; 95% CI, 1.07–1.78; *p* = 0.012) (Figure 1c,d; Table 1). The association between *PODXL2* expression and OS remained significant after adjustment for age, sex, and ELN 2022 adverse-risk status in both TCGA-LAML (HR, 1.67; 95% CI, 1.14–2.47; *p* = 0.009) and BeatAML (HR, 1.32; 95% CI, 1.01–1.71; *p* = 0.042) (Figure 1e,f; Table 1). Age and ELN 2022 adverse-risk status were also independently associated with OS in both cohorts, whereas sex was not independently associated with OS after multivariable adjustment. Formal testing of the proportional-hazards assumption showed no significant global evidence of non-proportionality in TCGA-LAML (global *p* = 0.281), whereas evidence of non-proportionality was observed in BeatAML (global *p* < 0.001; Supplementary Table S1). In a BeatAML sensitivity model incorporating time-dependent effects, the *PODXL2*-by-time interaction did not reach statistical significance (*p* = 0.067), while the estimated HR for *PODXL2*-high versus *PODXL2*-low remained above 1 across the evaluated follow-up times (Supplementary Table S2).

To further evaluate whether *PODXL2* provided prognostic information beyond major molecular risk markers, additional multivariable Cox regression models were constructed incorporating age, sex, WBC count, *NPM1* mutation, and *FLT3*-ITD (Supplementary Figure S1; Supplementary Table S3). *PODXL2*-high expression remained independently associated with inferior OS in both TCGA-LAML (HR, 1.80; 95% CI, 1.22–2.65; *p* = 0.003) and BeatAML (HR, 1.36; 95% CI, 1.03–1.80; *p* = 0.030). After additional adjustment for *TP53*, *FLT3*-ITD, and *NPM1* mutation, *PODXL2*-high expression remained significantly associated with inferior OS in TCGA-LAML (HR, 1.52; 95% CI, 1.02–2.27; *p* = 0.040) and demonstrated a similar effect size in BeatAML (HR, 1.29; 95% CI, 0.99–1.68; *p* = 0.055) (Supplementary Table S3). These findings support the prognostic value of *PODXL2* beyond established clinical and molecular risk factors.

Time-dependent ROC analyses demonstrated modest discriminatory performance of continuous *PODXL2* expression in both cohorts. In TCGA-LAML, the AUC values were 0.62, 0.63, and 0.64 at 12, 24, and 36 months, respectively, whereas in BeatAML the corresponding AUC values were 0.60, 0.59, and 0.58 (Figure 1g,h). Given the preferential representation of APL among *PODXL2*-low cases, we additionally performed a sensitivity analysis excluding APL while retaining the original *PODXL2* expression groups. In TCGA-LAML, *PODXL2*-high expression remained associated with inferior OS after exclusion of APL (*n* = 137; HR, 1.59; 95% CI, 1.07–2.35; *p* = 0.021; log-rank *p* = 0.020). In BeatAML, the association remained directionally consistent but did not reach statistical significance (*n* = 426; HR, 1.27; 95% CI, 0.99–1.64; *p* = 0.063; log-rank *p* = 0.062) (Supplementary Figure S2).

Collectively, these findings indicate that elevated *PODXL2* expression is consistently associated with inferior OS across two independent AML cohorts, with associations that persisted after adjustment for established clinical risk factors and were attenuated following additional molecular adjustment.

### 3.2. PODXL2-High AML Is Enriched for Adverse-Risk Genomic Alterations

We next investigated the association between *PODXL2* expression and recurrent AML-associated genomic alterations. Oncoprint analyses demonstrated differences in the mutational profiles of *PODXL2*-low and *PODXL2*-high AML in both the TCGA-LAML and BeatAML cohorts (Figure 2a,b).

In TCGA-LAML, *TP53* mutations were nominally enriched in *PODXL2*-high AML (13.2% vs. 1.3%; OR, 11.08; nominal *p* = 0.009), whereas *IDH2* mutations were nominally more frequent in *PODXL2*-low AML (16.0% vs. 5.3%; OR, 0.29; nominal *p* = 0.037) (Figure 2c,e). Similar patterns were observed in BeatAML, where *TP53* (12% vs. 5%; OR, 2.47; nominal *p* = 0.015) and *ASXL1* (14% vs. 4%; OR, 4.36; nominal *p* < 0.001) mutations were enriched in *PODXL2*-high AML, whereas *IDH2* mutations were more frequent in *PODXL2*-low AML (18% vs. 6%; OR, 0.29; nominal *p* < 0.001) (Figure 2d,f). Following BH correction for multiple testing, associations involving *TP53* and *IDH2* remained statistically significant in both cohorts, while the association between *ASXL1* mutation and *PODXL2*-high AML remained significant in BeatAML (Figure 2c,d). Overall, these findings demonstrate that elevated *PODXL2* expression is associated with recurrent genomic alterations linked to adverse-risk AML, particularly *TP53* and *ASXL1* mutations, and is relatively depleted in *IDH2*-mutated disease.

### 3.3. PODXL2 Expression Is Associated with Adverse-Risk Clinical and Cytogenetic Features in AML

We next examined associations between *PODXL2* expression and established AML risk-classification systems. In both TCGA-LAML and BeatAML, *PODXL2*-high AML was significantly enriched for ELN 2022 adverse-risk disease, whereas favorable-risk *AML* was more frequently represented among *PODXL2*-low cases (both *p* < 0.001; Figure 3a,b). Consistent with these findings, analysis of continuous *PODXL2* expression demonstrated significant differences across ELN 2022 risk categories in both cohorts, with higher expression observed in adverse-risk AML and markedly lower expression in favorable-risk AML (both *p* < 0.001; Figure 3c,d).

Within TCGA-LAML, *PODXL2* expression was also associated with cytogenetic-risk classification. *PODXL2*-high AML was enriched for poor-risk cytogenetics (*p* = 0.003; Figure 3e), and continuous *PODXL2* expression differed significantly across cytogenetic-risk groups (*p* = 0.005; Figure 3f). Although comparable cytogenetic-risk classifications were not available in BeatAML, concordant findings were observed for complex karyotypes, which were significantly more frequent in *PODXL2*-high AML in both TCGA-LAML (21.1% vs. 2.7%; *p* < 0.001) and BeatAML (18.7% vs. 7.3%; *p* < 0.001) (Table 2).

Additional demographic and clinical characteristics according to *PODXL2* expression status are summarized in Table 2. Taken together, these findings demonstrate a reproducible association between elevated *PODXL2* expression and adverse-risk disease biology across independent AML cohorts.

### 3.4. PODXL2 Expression Is Associated with Distinct FAB Morphology and Malignant Cell-State Signatures

We next evaluated the relationship between *PODXL2* expression and FAB classification. FAB subtype distributions differed significantly between *PODXL2*-low and *PODXL2*-high AML in both TCGA-LAML and BeatAML cohorts (both *p* < 0.001; Figure 4a,c). Continuous *PODXL2* expression also differed significantly across FAB subtypes in both cohorts (both *p* < 0.001; Figure 4b,d). Notably, FAB M3, corresponding to APL, exhibited among the lowest *PODXL2* expression levels across FAB subtypes, consistent with the preferential representation of APL among *PODXL2*-low cases.

To determine whether these morphologic differences were accompanied by differences in inferred leukemic differentiation-state programs, we analyzed malignant cell-state signature scores available in BeatAML. *PODXL2*-high AML exhibited significantly lower monocyte-like (*p* = 0.012, *q* = 0.035) and cDC-like cDC-like (*p* = 0.002, *q* = 0.013) malignant cell-state signature scores compared with *PODXL2*-low AML after multiple-testing correction (Figure 4e). The progenitor-like signature score was nominally higher in *PODXL2*-high AML (*p* = 0.048), although this difference did not remain significant after FDR adjustment (*q* = 0.096). HSC-like, GMP-like, and promonocyte-like signature scores were not significantly different between groups. FAB subtypes demonstrated distinct associations with malignant cell-state signature scores, as assessed by point-biserial correlation analysis (Figure 4f).

Together, these findings indicate that elevated *PODXL2* expression is associated with distinct morphologic features and differences in inferred malignant differentiation-state transcriptional signatures in AML.

### 3.5. PODXL2-High AML Displays Distinct Transcriptional and Regulatory Programs

To characterize the biological programs associated with *PODXL2* expression, transcriptomic analyses were performed independently in TCGA-LAML and BeatAML. Differential gene-expression analysis identified extensive transcriptional differences between *PODXL2*-low and *PODXL2*-high AML in both cohorts (Figure 5a).

Hallmark GSEA identified concordant enrichment of proliferative and metabolic programs in *PODXL2*-high AML across both cohorts (Figure 5b). Notably, MYC Targets V1/V2, E2F Targets, G2M Checkpoint, and DNA Repair were among the most consistently enriched pathways. Additional enriched pathways included Oxidative Phosphorylation, Unfolded Protein Response, mTORC1 Signaling, and multiple metabolic programs. Conversely, inflammatory-response and complement-related pathways were negatively enriched in *PODXL2*-high AML. Together, these findings indicate a reproducible transcriptional phenotype associated with elevated *PODXL2* expression.

To determine whether these pathway associations extended across the continuous range of *PODXL2* expression at the individual-sample level, we next performed GSVA followed by Spearman rank correlation analysis (Figure 5c). In both TCGA-LAML and BeatAML, higher *PODXL2* expression was positively associated with MYC/E2F-related, DNA-repair, cell-cycle, oxidative phosphorylation, mTORC1, and metabolic programs. In contrast, inflammatory-response and complement-related pathways demonstrated negative associations with *PODXL2* expression. These sample-level analyses recapitulated the major GSEA findings across the continuous spectrum of *PODXL2* expression in both cohorts.

We next used VIPER to determine whether *PODXL2*-associated transcriptional programs were accompanied by differences in inferred transcription-factor activity (Figure 5d). In both TCGA-LAML and BeatAML, elevated *PODXL2* expression was associated with increased inferred activity of several shared transcriptional regulators, including MYC, E2F2, TFDP1, MAZ, and SNAPC4. These cross-cohort findings provide complementary evidence for a shared regulatory state associated with elevated *PODXL2* expression.

Collectively, these analyses provide convergent transcriptomic evidence that elevated *PODXL2* expression is associated with a MYC/E2F-related proliferative transcriptional state, enhanced DNA-repair and metabolic programs, reduced inflammatory signaling, and distinct transcription-factor activity patterns across the TCGA-LAML and BeatAML cohorts.

### 3.6. PODXL2 Expression Is Associated with Differential Ex Vivo Drug Responses

To explore potential therapeutic implications of *PODXL2* expression, *ex vivo* drug-response data from BeatAML were evaluated. The overall drug-response landscape revealed multiple *PODXL2*-associated differences in *ex vivo* inhibitor response (Figure 6a,b). *PODXL2*-high AML exhibited higher scaled AUC values, consistent with reduced sensitivity, for OTX-015 (*p* = 0.001, *q* = 0.058), saracatinib (*p* = 0.001, *q* = 0.058), dasatinib (*p* = 0.003, *q* = 0.084), YM-155 (*p* = 0.002, *q* = 0.084), and regorafenib (*p* = 0.004, *q* = 0.098), all of which met the prespecified exploratory threshold of *q* < 0.10 (Figure 6c). Continuous-expression analyses yielded largely concordant findings. Higher *PODXL2* expression was associated with reduced sensitivity to OTX-015, saracatinib, YM-155, and regorafenib across the continuous range of *PODXL2* expression (Figure 6d), supporting the observations obtained using dichotomized *PODXL2* expression groups.

Taken together, these exploratory analyses identify *ex vivo* drug-response patterns associated with *PODXL2* expression. Because *PODXL2*-high AML differs in genomic and differentiation-state composition, these associations may partly reflect the broader biological characteristics of *PODXL2*-high AML and should be considered hypothesis-generating pending independent validation.

## 4. Discussion

In this study, we demonstrate that elevated *PODXL2* expression is associated with a clinically and biologically distinct high-risk AML state. Across two independent cohorts, *PODXL2*-high AML was associated with inferior survival, adverse ELN 2022 risk classification, poor-risk cytogenetics, and enrichment of adverse-risk molecular features.

Importantly, the association between elevated *PODXL2* expression and inferior survival persisted after adjustment for age, sex, and ELN 2022 risk status. However, the association was attenuated following additional molecular adjustment, particularly in BeatAML. These findings support *PODXL2* expression as a marker associated with adverse clinical outcome, although its incremental prognostic value beyond established clinical and molecular risk factors may be modest. Notably, analyses across the continuous range of *PODXL2* expression generally supported the categorical findings for survival, clinical and morphologic features, transcriptional pathway activity, and *ex vivo* drug response, indicating that the observed associations were not soley dependent on cohort-specific median dichotomization. A major finding of this study was the remarkable consistency of *PODXL2*-associated transcriptional programs across the TCGA-LAML and BeatAML cohorts. *PODXL2*-high AML demonstrated enrichment of MYC and E2F target programs together with G2M checkpoint, DNA repair, oxidative phosphorylation, mTORC1 signaling, unfolded protein response, fatty-acid metabolism, and peroxisome pathways. These findings were broadly reproduced across both pathway-enrichment and sample-level analyses and were accompanied by increased inferred activity of MYC-, E2F2-, TFDP1-, and MAZ-associated regulatory programs. Taken together, these observations identify a reproducible MYC/E2F-associated proliferative and metabolically active transcriptional state associated with elevated *PODXL2* expression.

The biological significance of these findings is supported by extensive evidence linking MYC and E2F signaling to leukemic proliferation, metabolism, cell-cycle progression, and stem-cell maintenance [34,35,36,37]. Concurrent enrichment of MYC targets, E2F targets, G2M checkpoint activity, and DNA-repair programs identifies a coordinated transcriptional state associated with increased proliferative activity and and adverse clinical outcomes in AML. Together, these findings suggest that proliferative and metabolic programs are interconnected components of a broader *PODXL2*-associated disease phenotype.

Although a direct mechanistic link between PODXL2 and MYC/E2F signaling has not been established in AML, experimental studies in solid tumors have implicated PODXL2 in Rac1/AKT- and PI3K/AKT-dependent signaling pathways that regulate cellular proliferation, survival, and stemness [20,21,22], providing a potential biological context for the MYC- and E2F-enriched transcriptional programs observed in *PODXL2*-high AML [38,39,40]. These observations provide biological context for our findings, while the present data support an association between elevated *PODXL2* expression and MYC/E2F-related transcriptional programs rather than a direct regulatory relationship.

Our genomic analyses provide additional insight into the *PODXL2*-high phenotype. *PODXL2*-high AML was enriched for *TP53*-mutated disease and relatively depleted of *IDH2*-mutated AML across both cohorts, while also exhibiting higher frequencies of poor-risk cytogenetic abnormalities. Given the established association between *TP53* alterations and adverse clinical outcomes [4], these findings further support the association of *PODXL2*-high AML with a high-risk disease state. However, most recurrent AML-associated mutations demonstrated limited associations with *PODXL2* expression, and the association between elevated *PODXL2* expression and inferior survival persisted after adjustment for established clinical risk factors, although it was attenuated after additional molecular adjustment, particularly in BeatAML. Together, these observations suggest that *PODXL2* may identify a broader expression-defined biological state rather than a discrete mutation-defined AML subtype.

Beyond its association with adverse-risk genomic and transcriptional features, *PODXL2* expression was also associated with distinct leukemic differentiation states. Notably, *PODXL2* expression was lowest in APL, whereas higher expression was observed across multiple non-APL AML subtypes. *PODXL2*-high AML also exhibited lower inferred monocyte-like and cDC-like malignant cell-state signature scores, supporting an association between *PODXL2* expression and transcriptionally inferred differentiation-state programs in AML. The progenitor-like signature score was nominally higher in *PODXL2*-high AML, although this difference did not remain significant following multiple-testing correction. HSC-like, GMP-like, and promonocyte-like signature scores were not significantly different between groups. Importantly, sensitivity analyses excluding APL showed that the association between *PODXL2*-high expression and inferior survival remained significant in TCGA-LAML and directionally consistent in BeatAML, although the latter did not reach statistical significance. These findings suggest that the survival association is not solely attributable to the inclusion of APL and further indicate that AML subtype and inferred differentiation-state programs may contribute to the biological and clinical features associated with *PODXL2* expression. 

An additional observation from this study was the association between *PODXL2* expression and *ex vivo* drug response. *PODXL2*-high AML exhibited reduced *ex vivo* sensitivity to several targeted agents, including the Bromodomain and Extra-Terminal (BET) inhibitor OTX-015 and SRC-family kinase inhibitors. Although these patterns were concordant across categorical and continuous *PODXL2* analyses, they should be considered exploratory. Because *PODXL2*-high AML differs substantially in genomic and differentiation-state composition, these drug-response associations may reflect the broader biological characteristics of *PODXL2*-high AML rather than a direct relationship between *PODXL2* expression and drug sensitivity. Of particular interest, *PODXL2*-high AML exhibited reduced *ex vivo* sensitivity to OTX-015, a BET inhibitor that targets MYC/E2F-associated transcriptional programs in AML and other hematologic malignancy models [41,42,43]. Given the concurrent enrichment of MYC/E2F-associated programs in *PODXL2*-high AML, this observation raises the possibility that the *PODXL2*-associated transcriptional state may be related to differential BET inhibitor response. However, this exploratory association does not establish a mechanistic relationship and requires further functional investigation. 

The consistent association between *PODXL2* expression and adverse outcome across both cohorts suggests that *PODXL2* may reflect aspects of AML disease biology associated with prognosis. However, the present analyses do not establish clear incremental predictive value beyond established risk variables. Several limitations should be acknowledged. This study was retrospective and based on publicly available datasets. Treatment information was not incorporated into the survival models, and differences in cohort composition, data availability, and molecular characteristics between TCGA-LAML and BeatAML may have contributed to cohort-specific variation in the observed associations. Pathway and transcription-factor activities were computationally inferred, and bulk transcriptomic analyses could not fully resolve the cellular origins of *PODXL2*-associated programs. Moreover, the present study did not include functional perturbation of *PODXL2*. Therefore, the observed transcriptional, proliferative, metabolic, and differentiation-associated phenotypes cannot be attributed directly to *PODXL2*. Functional studies in appropriate AML models will be required to determine whether *PODXL2* contributes directly to these biological states. Because *PODXL2* expression was also associated with AML differentiation and genetic features, residual confounding by differentiation state and genetic subtype cannot be excluded. The *ex vivo* drug-response findings are exploratory, were derived from a single cohort, and require further validation. In addition, *PODXL2*-low and *PODXL2*-high groups were defined using cohort-specific median expression values to facilitate within-cohort comparisons. These classifications do not represent a clinically validated or transferable expression cutoff. Establishing a reproducible *PODXL2* threshold for clinical application will require harmonized expression measurements and prospective validation.

## 5. Conclusions

In summary, elevated *PODXL2* expression is associated with inferior survival, adverse clinical and genomic features, altered differentiation-state programs, and MYC/E2F-associated proliferative and metabolic transcriptional programs. The broadly consistent associations observed across TCGA-LAML and BeatAML support the reproducibility of the *PODXL2*-associated phenotype across independent retrospective datasets. Collectively, these findings support *PODXL2* as a potential marker of an adverse AML-associated biological state, while its incremental clinical value beyond established risk-classification systems remains to be established. Future functional and prospective studies are warranted to clarify the biological role and clinical utility of *PODXL2* in AML.

## Figures and Tables

**Figure 1:**
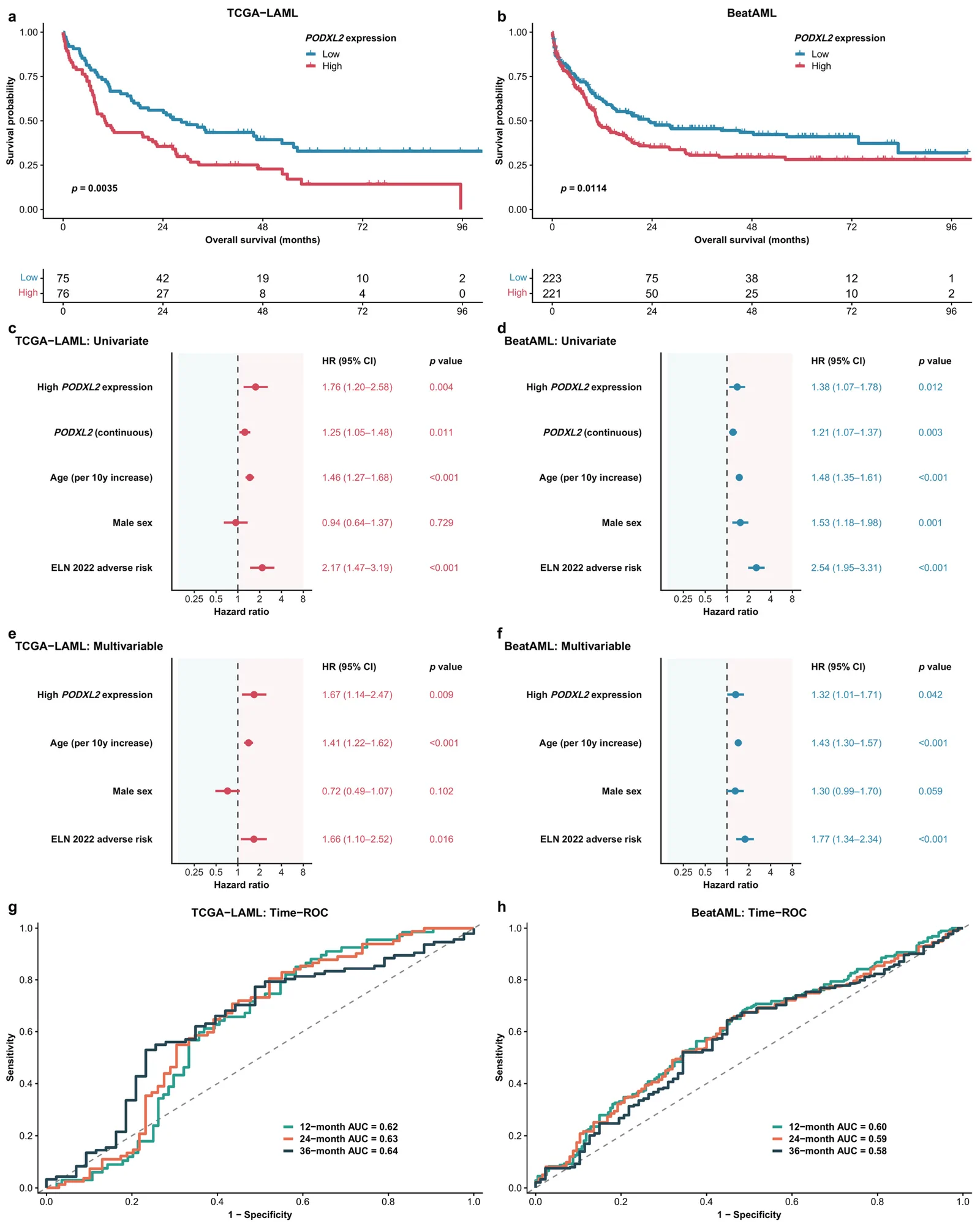
*PODXL2* expression is associated with inferior survival in TCGA-LAML and BeatAML. (**a**,**b**) Kaplan-Meier analysis of OS according to *PODXL2* expression status in TCGA-LAML (*n* = 151) and BeatAML (*n* = 444). (**c**,**d**) Univariable Cox regression analyses of OS in TCGA-LAML and BeatAML. In TCGA-LAML, all models included 151 patients. In BeatAML, evaluable sample sizes were *n* = 444 for categorical and continuous *PODXL2* expression and sex, *n* = 442 for age, and *n* = 432 for ELN 2022 adverse-risk status. (**e**,**f**) Multivariable Cox regression analyses of OS in TCGA-LAML (*n* = 151) and BeatAML (*n* = 430). (**g**,**h**) Time-dependent ROC analyses evaluating the prognostic performance of continuous *PODXL2* expression for 12-, 24-, and 36-month OS in TCGA-LAML (*n* = 151) and BeatAML (*n* = 444). Abbreviations: AUC, area under the curve; CI, confidence interval; ELN, European LeukemiaNet; HR, hazard ratio; OS, overall survival; ROC, receiver operating characteristic; SD, standard deviation.

**Figure 2:**
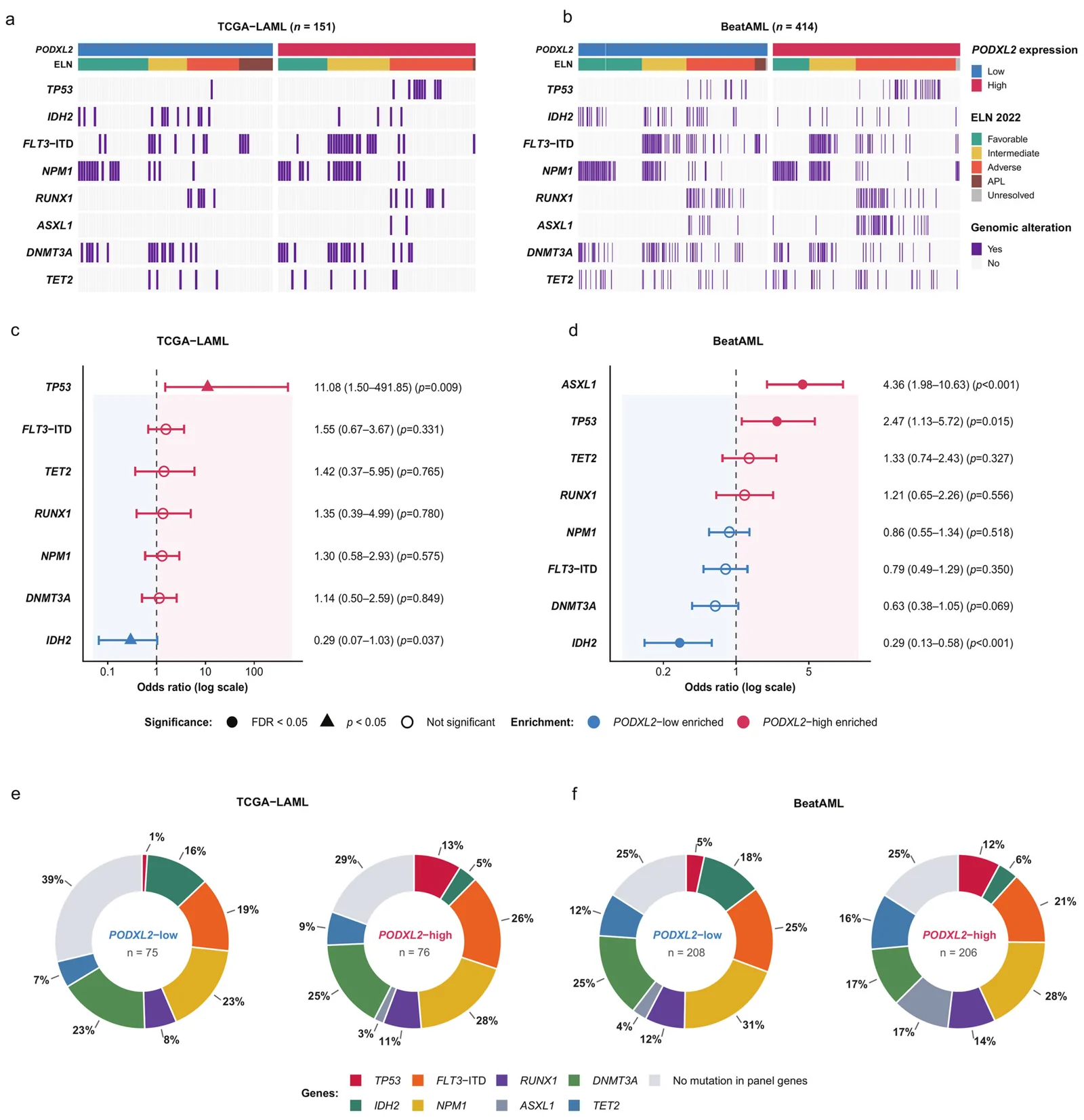
*PODXL2* expression is associated with distinct genomic alterations in AML. (**a**,**b**) Oncoprints showing recurrent AML-associated genomic alterations according to *PODXL2* expression status in TCGA-LAML and BeatAML. Samples are annotated by ELN 2022 risk category. (**c**,**d**) Associations between recurrent genomic alterations and *PODXL2* expression status in TCGA-LAML and BeatAML. OR > 1 indicates enrichment in *PODXL2*-high AML, whereas OR < 1 indicates enrichment in *PODXL2*-low AML. (**e**,**f**) Mutation-frequency profiles according to *PODXL2* expression status in TCGA-LAML and BeatAML. Genomic analyses included 151 TCGA-LAML patients (*PODXL2*-low, *n* = 75; *PODXL2*-high, *n* = 76) and 414 BeatAML patients with evaluable WES calls (*PODXL2*-low, *n* = 208; *PODXL2*-high, *n* = 206). Nominal *p*-values were derived from two-sided Fisher’s exact tests, and FDR-adjusted *q*-values were calculated using the BH procedure. Abbreviations: AML, acute myeloid leukemia; BH, Benjamini-Hochberg; CI, confidence interval; ELN, European LeukemiaNet; FDR, false discovery rate; OR, odds ratio; WES, whole-exome sequencing.

**Figure 3:**
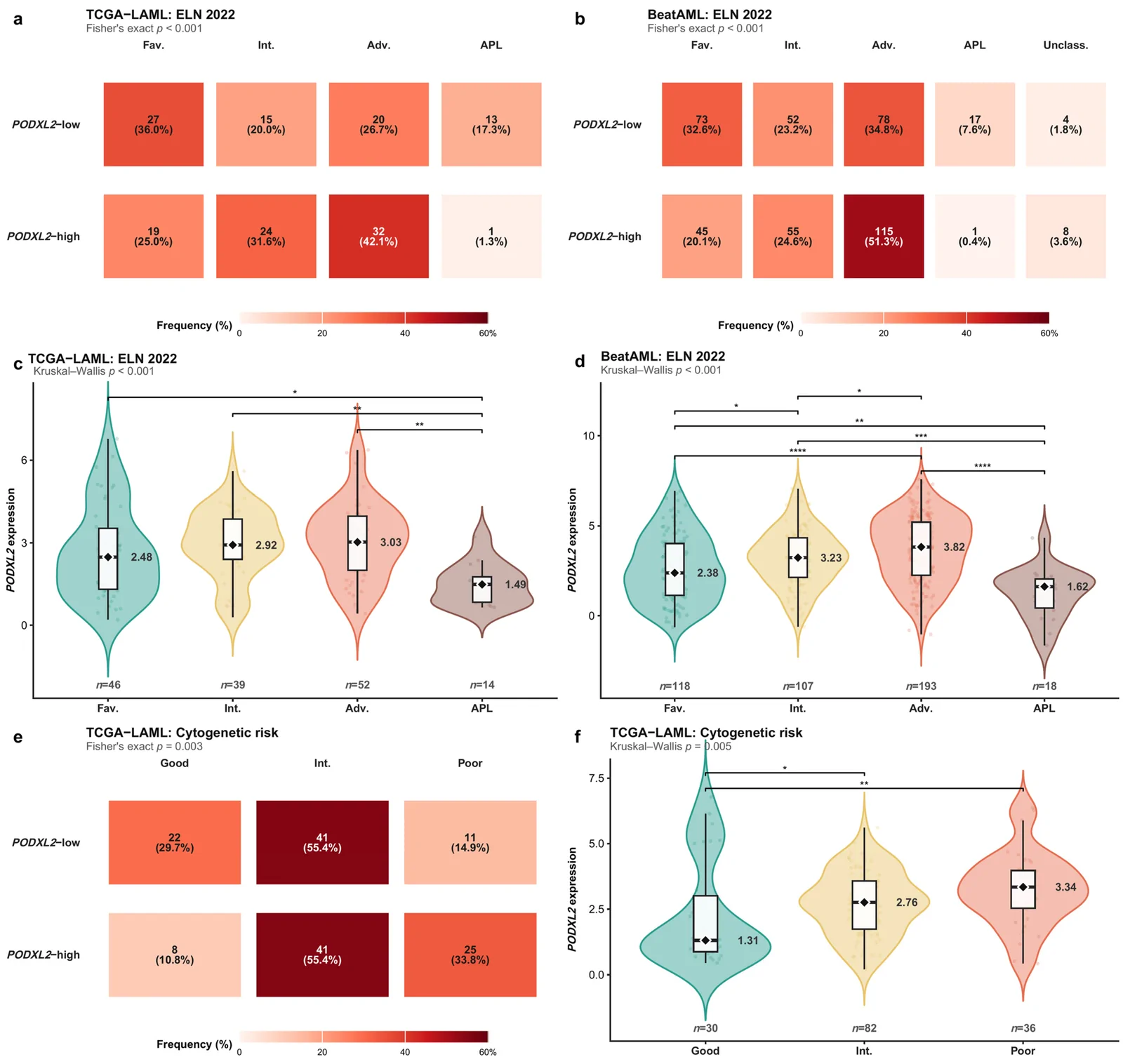
*PODXL2* expression is associated with adverse-risk clinical features in AML. (**a**,**b**) Distribution of ELN 2022 risk categories according to *PODXL2* expression status in TCGA-LAML and BeatAML. (**c**,**d**) *PODXL2* expression across ELN 2022 risk categories in TCGA-LAML and BeatAML. (**e**) Distribution of cytogenetic-risk categories according to *PODXL2* expression status in TCGA-LAML. (**f**) *PODXL2* expression across cytogenetic-risk categories in TCGA-LAML. Evaluable sample sizes were *n* = 151 for TCGA-LAML ELN 2022 analyses (**a**,**c**), *n* = 448 for the BeatAML ELN 2022 frequency analysis (**b**), *n* = 436 for the BeatAML *PODXL2* expression analysis across classified ELN 2022 categories (**d**), and *n* = 148 for TCGA-LAML cytogenetic-risk analyses (**e**,**f**). Significant pairwise comparisons are indicated as * adjusted *p* < 0.05, ** adjusted *p* < 0.01, *** adjusted *p* < 0.001, and **** adjusted *p* < 0.0001. Abbreviations: APL, acute promyelocytic leukemia; ELN, European LeukemiaNet.

**Figure 4:**
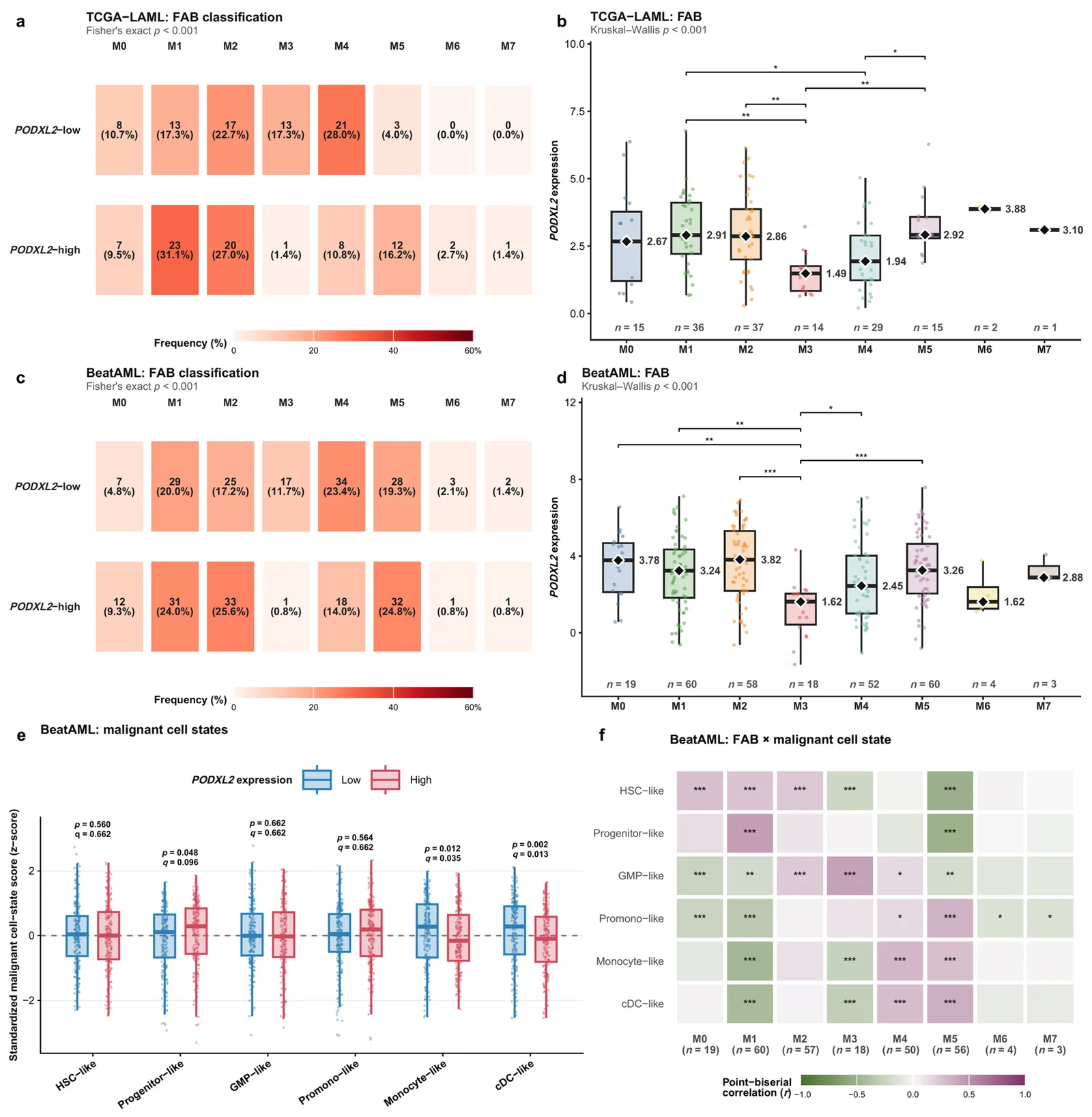
*PODXL2* expression is associated with distinct FAB morphology and malignant cell-state signatures. (**a**,**c**) Distribution of FAB categories according to *PODXL2* expression status in TCGA-LAML and BeatAML. (**b**,**d**) *PODXL2* expression across FAB categories in TCGA-LAML and BeatAML. (**e**) Malignant cell-state signature scores according to *PODXL2* expression status in BeatAML. (**f**) Associations between FAB categories and malignant cell-state signatures in BeatAML. Evaluable sample sizes were *n* = 149 for TCGA-LAML FAB analyses (**a**,**b**), *n* = 274 for BeatAML FAB analyses (**c**,**d**), *n* = 433 for the BeatAML malignant cell-state comparison (**e**), and *n* = 267 for the FAB–malignant cell-state analysis (**f**). Significant pairwise comparisons are indicated as * adjusted *p* < 0.05, ** adjusted *p* < 0.01, and *** adjusted *p* < 0.001. Abbreviations: cDC, conventional dendritic cell; FAB, French-American-British; GMP, granulocyte-monocyte progenitor; HSC, hematopoietic stem cell.

**Figure 5:**
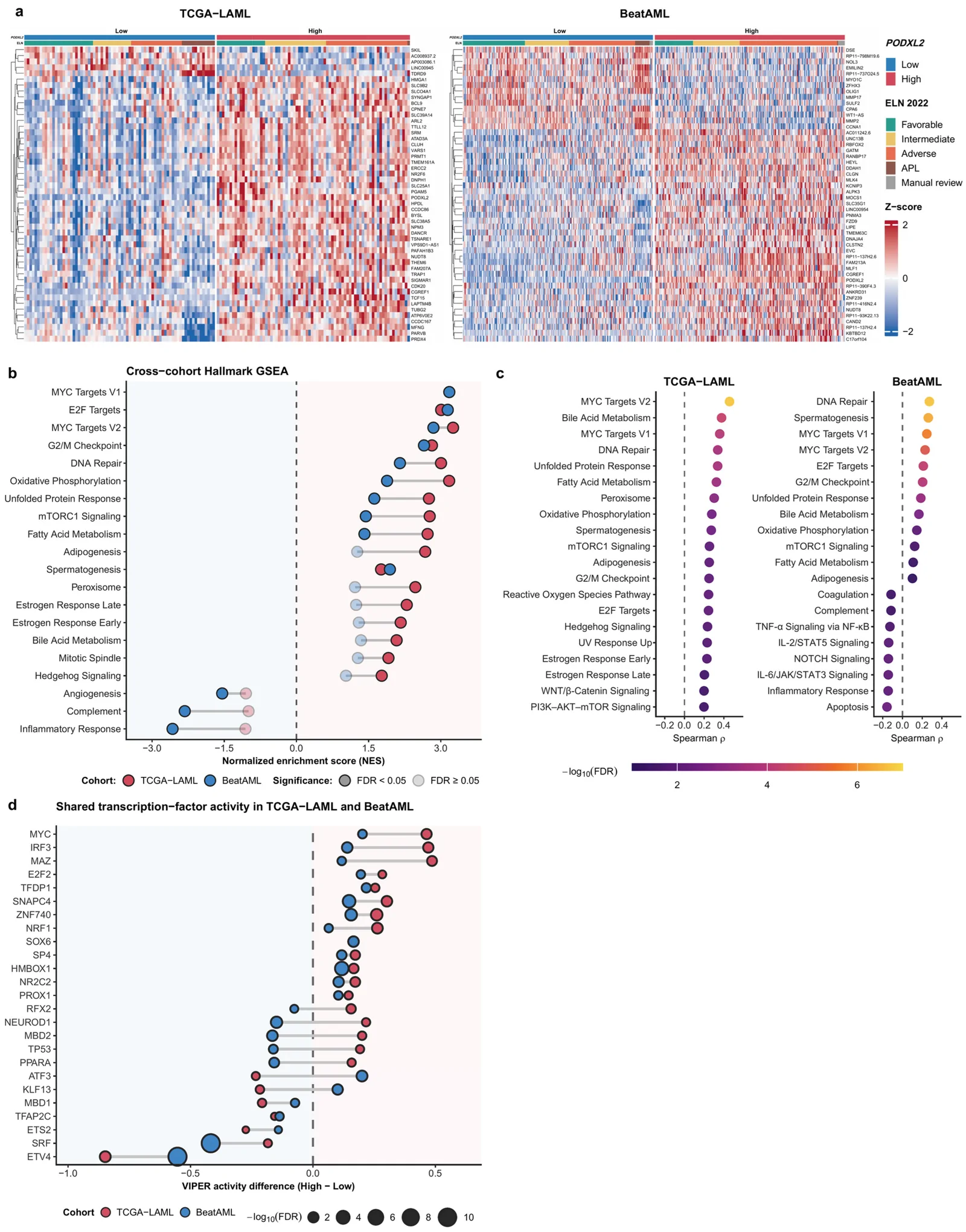
*PODXL2*-high AML exhibits distinct transcriptomic and regulatory programs. (**a**) Differential gene-expression profiles associated with *PODXL2* expression in TCGA-LAML and BeatAML. (**b**) Hallmark GSEA comparing *PODXL2*-low and *PODXL2*-high AML. Positive NES values indicate enrichment in *PODXL2*-high AML. (**c**) Associations between continuous *PODXL2* expression and Hallmark pathway activity in TCGA-LAML and BeatAML. (**d**) Differential TF activity associated with *PODXL2* expression inferred using DoRothEA and VIPER. Transcriptomic analyses included 151 TCGA-LAML samples (*PODXL2*-low, *n* = 75; *PODXL2*-high, *n* = 76) and 448 BeatAML samples (*PODXL2*-low, *n* = 224; *PODXL2*-high, *n* = 224). Abbreviations: AML, acute myeloid leukemia; DoRothEA, Discriminant Regulon Expression Analysis; FDR, false discovery rate; GSEA, gene-set enrichment analysis; GSVA, gene-set variation analysis; NES, normalized enrichment score; TF, transcription factor; VIPER, Virtual Inference of Protein-activity by Enriched Regulon analysis.

**Figure 6:**
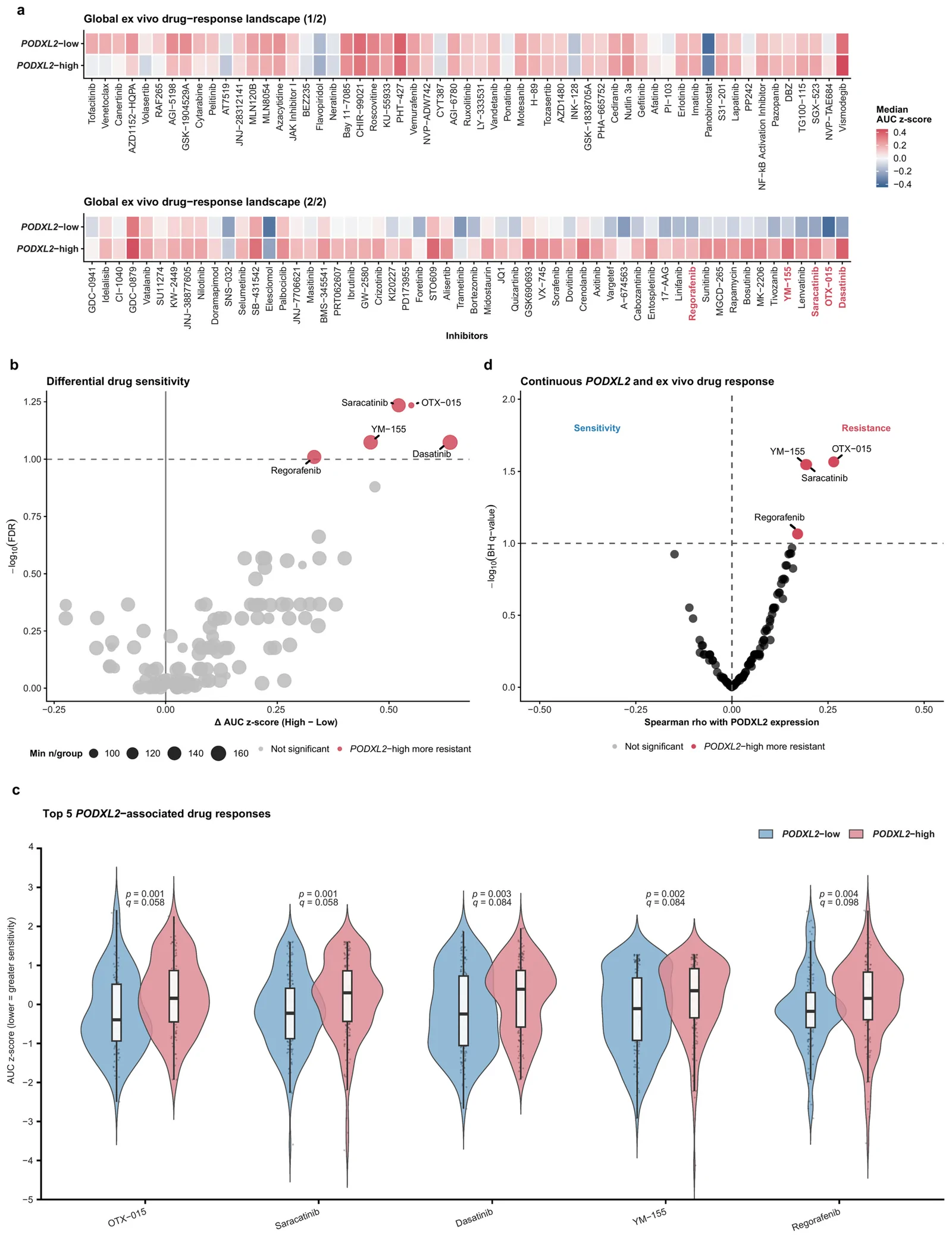
*PODXL2* expression is associated with differential *ex vivo* drug responses in BeatAML. (**a**) Global *ex vivo* drug-response landscape according to *PODXL2* expression status. (**b**) Differential drug response between *PODXL2*-high and *PODXL2*-low AML. Positive values indicate greater reduced sensitivity in *PODXL2*-high AML. (**c**) Drug-response distributions for the top *PODXL2*-associated compounds. (**d**) Associations between continuous *PODXL2* expression and drug response. *Ex vivo* drug-response analyses included 344 BeatAML samples with available drug-response data (*PODXL2*-low, *n* = 165; *PODXL2*-high, *n* = 179) across 113 evaluated compounds. Because drug-response availability varied across compounds, sample sizes were drug-specific. For the five compounds displayed in panel (**c**), evaluable sample sizes were OTX-015, *n* = 188; saracatinib, *n* = 299; dasatinib, *n* = 324; YM-155, *n* = 305; and regorafenib, *n* = 299. Drug-specific sample sizes were also used for the continuous *PODXL2*–drug-response correlations in panel (**d**). Abbreviations: AML, acute myeloid leukemia; AUC, area under the curve; BH, Benjamini-Hochberg; FDR, false discovery rate.

**Table 1:** Univariable and multivariable Cox proportional-hazards regression analyses of overall survival in TCGA-LAML and BeatAML.

Variable	Univariable HR (95% CI)	*p*-Value	Multivariable HR (95% CI)	*p*-Value
TCGA-LAML				
*PODXL2*-high vs. low	1.76 (1.20–2.58)	0.004	1.67 (1.14–2.47)	0.009
*PODXL2* expression, per 1-SD increase	1.25 (1.05–1.48)	0.011	—	—
Age, per 10-year increase	1.46 (1.27–1.68)	<0.001	1.41 (1.22–1.62)	<0.001
Male sex	0.94 (0.64–1.37)	0.729	0.72 (0.49–1.07)	0.102
ELN 2022 adverse risk	2.17 (1.47–3.19)	<0.001	1.66 (1.10–2.52)	0.016
BeatAML				
*PODXL2*-high vs. low	1.38 (1.07–1.78)	0.012	1.32 (1.01–1.71)	0.042
*PODXL2* expression, per 1-SD increase	1.21 (1.07–1.37)	0.003	—	—
Age, per 10-year increase	1.48 (1.35–1.61)	<0.001	1.43 (1.31–1.57)	<0.001
Male sex	1.53 (1.18–1.98)	0.001	1.30 (0.99–1.70)	0.059
ELN 2022 adverse risk	2.54 (1.95–3.31)	<0.001	1.77 (1.34–2.34)	<0.001

Data are presented as HRs with 95% CIs. Patients were stratified into *PODXL2*-high and *PODXL2*-low groups using the cohort-specific median *PODXL2* expression. *p*-values are two-sided. The multivariable model included *PODXL2* expression group (high vs. low), age (per 10-year increase), sex, and ELN 2022 adverse-risk status. Continuous *PODXL2* expression was standardized within each cohort and evaluated per 1-SD increase in univariable analysis only. Reference categories were *PODXL2*-low, female sex, and non-adverse ELN 2022 risk. Abbreviations: HR, hazard ratio; CI, confidence interval; ELN, European LeukemiaNet; SD, standard deviation.

**Table 2:** Baseline clinical, disease, and molecular characteristics according to PODXL2 expression in TCGA-LAML and BeatAML.

Variable	TCGA-LAML				BeatAML			
	Overall	*PODXL2*-Low	*PODXL2*-High	*p*-Value	Overall	*PODXL2*-Low	*PODXL2*-High	*p*-Value
Demographic characteristics								
Total patients, *N*	151	75	76		448	224	224	
Age at diagnosis, median (range)	56.0 (21.0–88.0)	55.0 (21.0–77.0)	57.5 (21.0–88.0)	0.728	61.0 (0.0–88.0)	61.0 (5.0–88.0)	62.0 (0.0–87.0)	0.483
Age >= 60 years, *n*/*N* (%)	67/151 (44.4%)	31/75 (41.3%)	36/76 (47.4%)	0.514	247/446 (55.4%)	121/223 (54.3%)	126/223 (56.5%)	0.703
Male sex, *n*/*N* (%)	83/151 (55.0%)	36/75 (48.0%)	47/76 (61.8%)	0.103	245/448 (54.7%)	108/224 (48.2%)	137/224 (61.2%)	0.008
Race, *n*/*N* (%)				0.670				0.496
White	133/151 (88.1%)	67/75 (89.3%)	66/76 (86.8%)		282/313 (90.1%)	142/156 (91.0%)	140/157 (89.2%)	
Black	13/151 (8.6%)	5/75 (6.7%)	8/76 (10.5%)		12/313 (3.8%)	5/156 (3.2%)	7/157 (4.5%)	
Asian	3/151 (2.0%)	2/75 (2.7%)	1/76 (1.3%)		10/313 (3.2%)	4/156 (2.6%)	6/157 (3.8%)	
Other	1/151 (0.7%)	0/75 (0.0%)	1/76 (1.3%)		5/313 (1.6%)	4/156 (2.6%)	1/157 (0.6%)	
Unknown	1/151 (0.7%)	1/75 (1.3%)	0/76 (0.0%)		4/313 (1.3%)	1/156 (0.6%)	3/157 (1.9%)	
Baseline disease characteristics								
WBC count, median (IQR)	17.9 (4.6–47.8)	22.2 (4.2–47.6)	15.0 (4.9–47.7)	0.759	25.5 (8.9–64.1)	26.9 (9.4–59.0)	25.0 (8.2–69.0)	0.968
Bone marrow blasts, %, median (IQR)	72.0 (52.0–85.0)	72.0 (53.5–86.5)	72.0 (52.0–83.2)	0.589	74.0 (47.0–88.0)	74.9 (50.0–88.0)	73.0 (40.0–88.0)	0.335
Peripheral blood blasts, %, median (IQR)	40.0 (10.0–67.0)	39.0 (10.0–68.0)	42.0 (7.8–63.2)	0.902	56.0 (23.2–80.0)	56.0 (25.8–83.0)	55.5 (22.2–79.0)	0.383
FAB classification, *n*/*N* (%)				<0.001				<0.001
M0	15/149 (10.1%)	8/75 (10.7%)	7/74 (9.5%)		19/274 (6.9%)	7/145 (4.8%)	12/129 (9.3%)	
M1	36/149 (24.2%)	13/75 (17.3%)	23/74 (31.1%)		60/274 (21.9%)	29/145 (20.0%)	31/129 (24.0%)	
M2	37/149 (24.8%)	17/75 (22.7%)	20/74 (27.0%)		58/274 (21.2%)	25/145 (17.2%)	33/129 (25.6%)	
M3	14/149 (9.4%)	13/75 (17.3%)	1/74 (1.4%)		18/274 (6.6%)	17/145 (11.7%)	1/129 (0.8%)	
M4	29/149 (19.5%)	21/75 (28.0%)	8/74 (10.8%)		52/274 (19.0%)	34/145 (23.4%)	18/129 (14.0%)	
M5	15/149 (10.1%)	3/75 (4.0%)	12/74 (16.2%)		60/274 (21.9%)	28/145 (19.3%)	32/129 (24.8%)	
M6	2/149 (1.3%)	0/75 (0.0%)	2/74 (2.7%)		4/274 (1.5%)	3/145 (2.1%)	1/129 (0.8%)	
M7	1/149 (0.7%)	0/75 (0.0%)	1/74 (1.4%)		3/274 (1.1%)	2/145 (1.4%)	1/129 (0.8%)	
Disease classification								
ELN 2022 risk category, *n*/*N* (%)				<0.001				<0.001
Favorable	46/151 (30.5%)	27/75 (36.0%)	19/76 (25.0%)		118/448 (26.3%)	73/224 (32.6%)	45/224 (20.1%)	
Intermediate	39/151 (25.8%)	15/75 (20.0%)	24/76 (31.6%)		107/448 (23.9%)	52/224 (23.2%)	55/224 (24.6%)	
Adverse	52/151 (34.4%)	20/75 (26.7%)	32/76 (42.1%)		193/448 (43.1%)	78/224 (34.8%)	115/224 (51.3%)	
APL	14/151 (9.3%)	13/75 (17.3%)	1/76 (1.3%)		18/448 (4.0%)	17/224 (7.6%)	1/224 (0.4%)	
Molecular and cytogenetic characteristics								
*TP53* mutation, *n*/*N* (%)	11/151 (7.3%)	1/75 (1.3%)	10/76 (13.2%)	0.009	37/448 (8.3%)	11/224 (4.9%)	26/224 (11.6%)	0.015
*FLT3*-ITD mutation, *n*/*N* (%)	34/151 (22.5%)	14/75 (18.7%)	20/76 (26.3%)	0.331	106/448 (23.7%)	59/224 (26.3%)	47/224 (21.0%)	0.221
*NPM1* mutation, *n*/*N* (%)	38/151 (25.2%)	17/75 (22.7%)	21/76 (27.6%)	0.575	125/448 (27.9%)	67/224 (29.9%)	58/224 (25.9%)	0.399
*RUNX1* mutation, *n*/*N* (%)	14/151 (9.3%)	6/75 (8.0%)	8/76 (10.5%)	0.780	53/448 (11.8%)	24/224 (10.7%)	29/224 (12.9%)	0.559
*ASXL1* mutation, *n*/*N* (%)	2/151 (1.3%)	0/75 (0.0%)	2/76 (2.6%)	0.497	44/448 (9.8%)	9/224 (4.0%)	35/224 (15.6%)	<0.001
*CEBPA* bZIP in-frame mutation, *n*/*N* (%)	8/151 (5.3%)	4/75 (5.3%)	4/76 (5.3%)	1.000	6/414 (1.4%)	2/208 (1.0%)	4/206 (1.9%)	0.448
Myelodysplasia-related gene mutation, *n*/*N* (%)	23/151 (15.2%)	10/75 (13.3%)	13/76 (17.1%)	0.652	155/416 (37.3%)	71/208 (34.1%)	84/208 (40.4%)	0.224
Complex karyotype, *n*/*N* (%)	18/151 (11.9%)	2/75 (2.7%)	16/76 (21.1%)	<0.001	54/414 (13.0%)	15/205 (7.3%)	39/209 (18.7%)	<0.001
Outcome								
Overall survival events, *n*/*N* (%)	108/151 (71.5%)	47/75 (62.7%)	61/76 (80.3%)	0.020	245/444 (55.2%)	110/223 (49.3%)	135/221 (61.1%)	0.013

Data are presented as *n*/*N* (%) or median (IQR), unless otherwise indicated. Age at diagnosis is presented as median (range). Continuous variables were compared using two-sided Wilcoxon rank-sum tests, and categorical variables using two-sided Fisher’s exact tests. Percentages are based on patients with available data for each variable. ELN 2022 categories include APL as a separate category. Abbreviations: AML, acute myeloid leukemia; APL, acute promyelocytic leukemia; BM, bone marrow; ELN, European LeukemiaNet; FAB, French-American-British; IQR, interquartile range; PB, peripheral blood; WBC, white blood cell.

## Data Availability

The data presented in this study are available in The Cancer Genome Atlas Program (TCGA)—NCI and https://www.ncbi.nlm.nih.gov/projects/gap/cgi-bin/study.cgi?study_id=phs001657.v2.p1 (accessed on 8 April 2026), reference number [31,32]. These data were derived from the following resources available in the public domain: UCSC Xena for Cancer Genomics.

## Supplementary Materials

The following supporting information can be downloaded at: https://www.mdpi.com/article/10.3390/cancers18193219/s1, Figure S1: Additional multivariable Cox regression analyses of overall survival according to *PODXL2* expression; Figure S2: Overall survival according to *PODXL2* expression after exclusion of acute promyelocytic leukemia; Table S1: Proportional-hazards assumption testing for the multivariable Cox regression models; Table S2: Time-varying Cox sensitivity analysis in BeatAML; Table S3: Additional multivariable Cox regression models incorporating recurrent AML-associated molecular alterations.
