# Supplementary material for: PODXL2 Expression Is Associated with Inferior Survival, Adverse-Risk Features, and Distinct Transcriptional Programs in Acute Myeloid Leukemia
Source: Cancers (Basel). 2026 Oct 6;18(19):3219. doi: 10.3390/cancers18193219 (PMC13664358; doi:10.3390/cancers18193219)
Supplement: Supplementary file 1 [file cancers-18-03219-s001.zip › cancers-4592493-supplementary.pdf]

Supplementary Table S1. Proportional-hazards assumption testing for the multivariable Cox regression models

Cohort	Variable	Chi-square	df	*p*-value
TCGA-LAML	*PODXL2*-high vs low	0.169	1	0.681
TCGA-LAML	Age, per 10-year increase	4.850	1	0.028
TCGA-LAML	Male sex	0.489	1	0.484
TCGA-LAML	ELN 2022 adverse risk	0.195	1	0.659
TCGA-LAML	Global test	5.060	4	0.281
BeatAML	*PODXL2*-high vs low	4.420	1	0.035
BeatAML	Age, per 10-year increase	6.370	1	0.012
BeatAML	Male sex	2.600	1	0.107
BeatAML	ELN 2022 adverse risk	4.990	1	0.026
BeatAML	Global test	19.300	4	<0.001

The proportional-hazards assumption was formally evaluated using tests based on scaled Schoenfeld residuals for the multivariable Cox regression models in TCGA-LAML (n = 151) and BeatAML (n = 430). p values are two-sided. Abbreviations: df, degrees of freedom; ELN, European LeukemiaNet.

Supplementary Table S2. Time-varying Cox sensitivity analysis in BeatAML

Time-dependent interaction terms

Variable	Coefficient	exp(coefficient)	SE	*p*-value
PODXL2-high × log(time)	0.1568	1.1697	0.0855	0.067
Age × log(time)	-0.0994	0.9053	0.0323	0.002
ELN 2022 adverse risk × log(time)	0.2763	1.3182	0.0879	0.002

Estimated *PODXL2*-high versus PODXL2-low hazard ratios over follow-up

Follow-up time, months	Estimated PODXL2-high vs low HR
6	1.40
12	1.56
24	1.74
36	1.85
60	2.01

The sensitivity analysis included 430 patients. Time-dependent interactions with log-transformed follow-up time were included for *PODXL2* expression status, age, and ELN 2022 adverse-risk status because these covariates showed evidence of non-proportional hazards. Estimated *PODXL2* hazard ratios at selected follow-up times were calculated from the fitted *PODXL2* main coefficient and *PODXL2* × log(time) interaction. Abbreviations: HR, hazard ratio; SE, standard error; ELN, European LeukemiaNet.

Supplementary Table S3. Additional multivariable Cox regression models incorporating recurrent AML-associated molecular alterations

Variable	TCGA-LAML HR (95% CI)	*P*-value	BeatAML HR (95% CI)	*p*-value
***PODXL2*-high vs low + age per 10 years + male sex + WBC per 2-fold increase + *NPM1* mutation + *FLT3*-ITD**				
PODXL2-high vs low	1.80 (1.22–2.65)	0.003	1.36 (1.03–1.80)	0.030
Age, per 10-year increase	1.56 (1.35–1.80)	<0.001	1.47 (1.34–1.61)	<0.001
Male sex	0.79 (0.54–1.16)	0.232	1.28 (0.96–1.70)	0.099
WBC, per 2-fold increase	1.18 (1.06–1.30)	0.001	1.11 (1.04–1.20)	0.004
NPM1 mutation	0.80 (0.50–1.29)	0.360	0.69 (0.49–0.96)	0.026
FLT3-ITD	1.63 (1.01–2.63)	0.046	1.04 (0.75–1.45)	0.809
***PODXL2*-high vs low + age per 10 years + male sex + *TP53* mutation + *FLT3*-ITD + *NPM1* mutation**				
PODXL2-high vs low	1.52 (1.02–2.27)	0.040	1.29 (0.99–1.68)	0.055
Age, per 10-year increase	1.46 (1.26–1.68)	<0.001	1.47 (1.34–1.60)	<0.001
Male sex	0.78 (0.53–1.14)	0.201	1.33 (1.01–1.74)	0.040
TP53 mutation	3.59 (1.76–7.33)	<0.001	2.80 (1.92–4.08)	<0.001
FLT3-ITD	1.72 (1.05–2.82)	0.031	1.18 (0.86–1.61)	0.300
NPM1 mutation	1.14 (0.71–1.83)	0.583	0.87 (0.64–1.18)	0.383

Data are presented as hazard ratios (HRs) with 95% confidence intervals (CIs). Patients were stratified into *PODXL2*-high and *PODXL2*-low groups using the cohort-specific median *PODXL2* expression. *p* values are two-sided. The first multivariable model included *PODXL2* expression group (high vs low), age (per 10-year increase), sex, WBC (per 2-fold increase), *NPM1* mutation, and *FLT3*-ITD. The second multivariable model included *PODXL2* expression group (high vs low), age (per 10-year increase), sex, *TP53* mutation, *FLT3*-ITD, and *NPM1* mutation. The evaluable sample sizes were *n* = 151 for both models in TCGA-LAML and *n* = 380 and *n* = 442 for the first and second models, respectively, in BeatAML. Reference categories were *PODXL2*-low, female sex, and absence of the indicated molecular alteration. Abbreviations: HR, hazard ratio; CI, confidence interval; WBC, white blood cell count; TCGA-LAML, The Cancer Genome Atlas acute myeloid leukemia cohort.

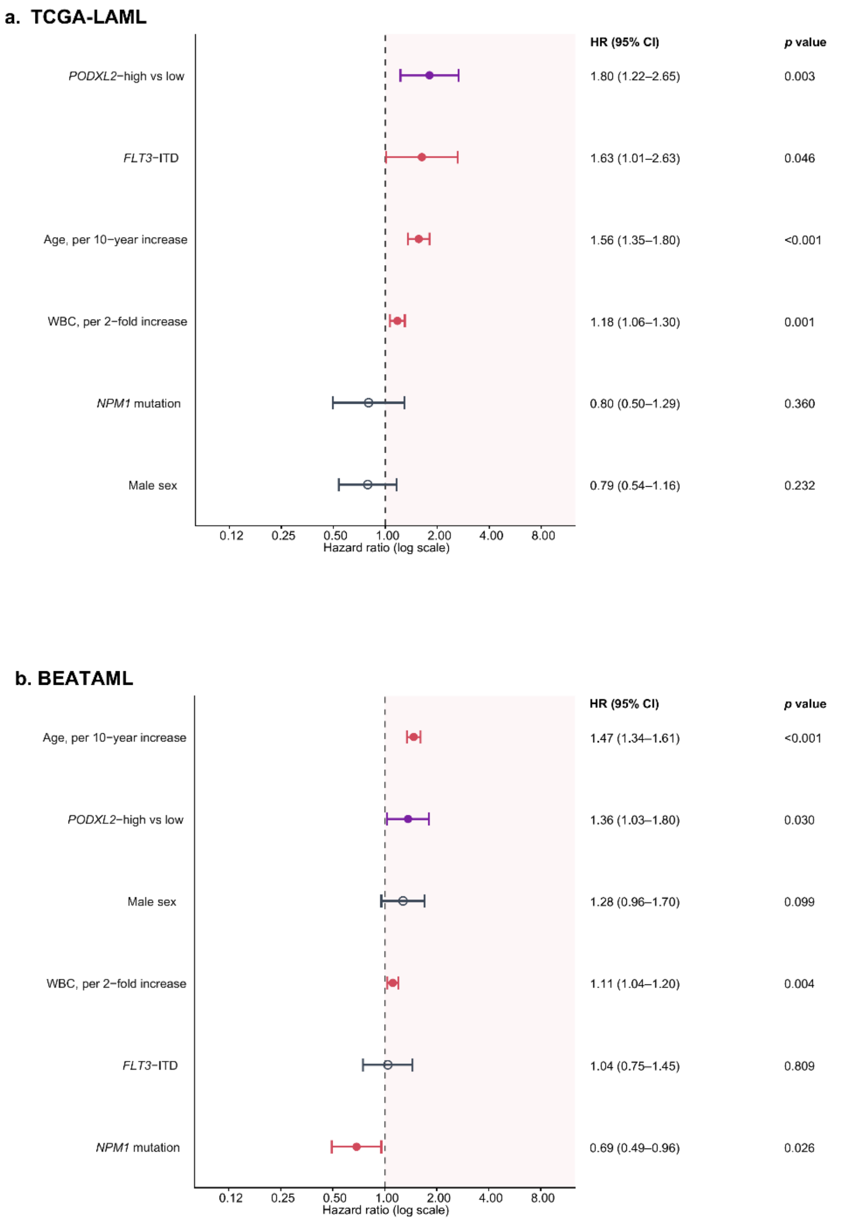

Supplementary Figure S1. Additional multivariable Cox regression analyses of overall survival according to *PODXL2* expression.

Forest plots showing multivariable Cox regression analyses of overall survival (OS) in (a) TCGA-LAML (*n* = 151) and (b) BeatAML (*n* = 380). Models included *PODXL2* expression status (high vs low), age (per 10-year increase), sex, WBC (per 2-fold increase), *NPM1* mutation status, and *FLT3*-ITD status. Points represent hazard ratios (HRs), and horizontal lines indicate 95% confidence intervals (CIs). *p* values are two-sided. Reference categories were *PODXL2*-low, female sex, and absence of the indicated molecular alteration. Abbreviations: OS, overall survival; HR, hazard ratio; CI, confidence interval; WBC, white blood cell count.

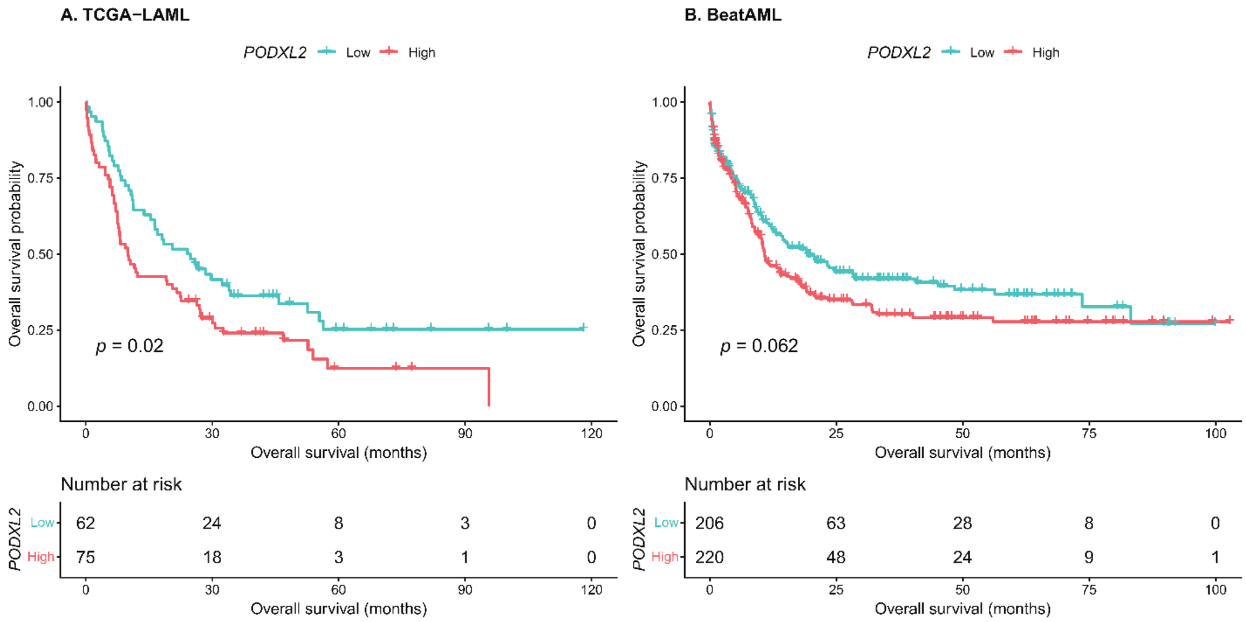

Supplementary Figure S2. Overall survival according to *PODXL2* expression after exclusion of acute promyelocytic leukemia. Kaplan–Meier analysis of overall survival according to the original cohort-defined *PODXL2*-high and *PODXL2*-low classifications after exclusion of acute promyelocytic leukemia (APL) in (A) TCGA-LAML and (B) BeatAML. *PODXL2* expression groups were retained from the primary analysis and were not redefined after APL exclusion. *p* values were calculated using two-sided log-rank tests.
